# Clinical outcomes in imported endothelium-in preloaded vs. surgeon-loaded DMEKs in Asian eyes

**DOI:** 10.3389/fmed.2025.1580733

**Published:** 2025-05-13

**Authors:** Ezekiel Ze Ken Cheong, Qiu Ying Wong, Howard Cajucom-Uy, Hla Myint Htoon, Marcus Ang

**Affiliations:** ^1^Ophthalmology and Visual Sciences Academic Clinical Program, Duke-NUS Medical School, Singapore, Singapore; ^2^Singapore National Eye Centre, Singapore, Singapore; ^3^Singapore Eye Research Institute, Singapore, Singapore

**Keywords:** Descemet membrane endothelial keratoplasty, preloaded grafts, pull-through, endothelium-in, intra-operative complications, post-operative complications

## Abstract

**Purpose:**

To perform a direct, prospective, comparative analysis of the complications associated with imported preloaded grafts versus surgeon-loaded grafts in Descemet membrane endothelial keratoplasty (DMEK) in Asian eyes.

**Methods:**

A total of 20 consecutive preloaded DMEKs were matched by donor age with 40 surgeon-loaded DMEKs for the indications of Fuchs’ endothelial cell dystrophy (FECD) and pseudophakic bullous keratopathy (PBK). All cases of preloaded and surgeon-loaded DMEKs were by a single surgeon in the Singapore National Eye Centre and utilized endothelium-in pull-through cartridges (CORONET DMEK Endoglide; Network Medical Products, United Kingdom). Imported preloaded grafts were prepared 72 to 96 h before surgery. The main outcome measures were intra-operative complications such as graft tears, extrusion, and high vitreous pressure. Secondary outcome measures were endothelial cell loss and early post-operative complications such as ocular hypertension, graft detachment, and rebubbling, up to 6 months post-operatively.

**Results:**

Preloaded DMEKs had significantly shorter intra-operative times (26.2 min vs. 39.5 min; *p* < 0.001) than surgeon-loaded DMEKs but were associated with increased intra-operative risk of conversion to standard injector-DMEK (15% vs. 0%; *p* = 0.033). However, there was no increase in overall intra-operative complications (40% vs. 22.5%; *p* = 0.156), early post-operative complications (35% vs. 30%; *p* = 0.772), and rebubbling rate (5% vs. 5%; *p* > 0.999). Visual outcomes and endothelial cell loss were not significantly different in both groups.

**Conclusion:**

In our Asian study cohort of pull-through DMEKs, endothelium-in preloaded DMEKs were significantly faster than surgeon-loaded DMEKs and had comparable clinical outcomes.

## Introduction

Corneal endothelial cells are crucial for maintaining corneal transparency but are terminally differentiated and non-regenerative (1, 2). Endothelial dysfunction is the most common indication for corneal transplantation (3). One such technique is Descemet membrane endothelial keratoplasty (DMEK), where the prepared graft consists only of the Descemet membrane and the endothelium (4). Compared to its predecessor technique of Descemet stripping automated endothelial keratoplasty (DSAEK), the graft is thinner, and the clinical outcomes are more favorable (5, 6), justifying its increasingly high adoption rate amongst cornea surgeons for cornea endothelium replacement (7). As with other keratoplasty techniques, DMEKs are dependent on donor tissue supply, which is limited in a small country such as Singapore. Imported preloaded DMEK grafts have emerged as a viable method of increasing the supply of donor tissue.

**Figure 1:**
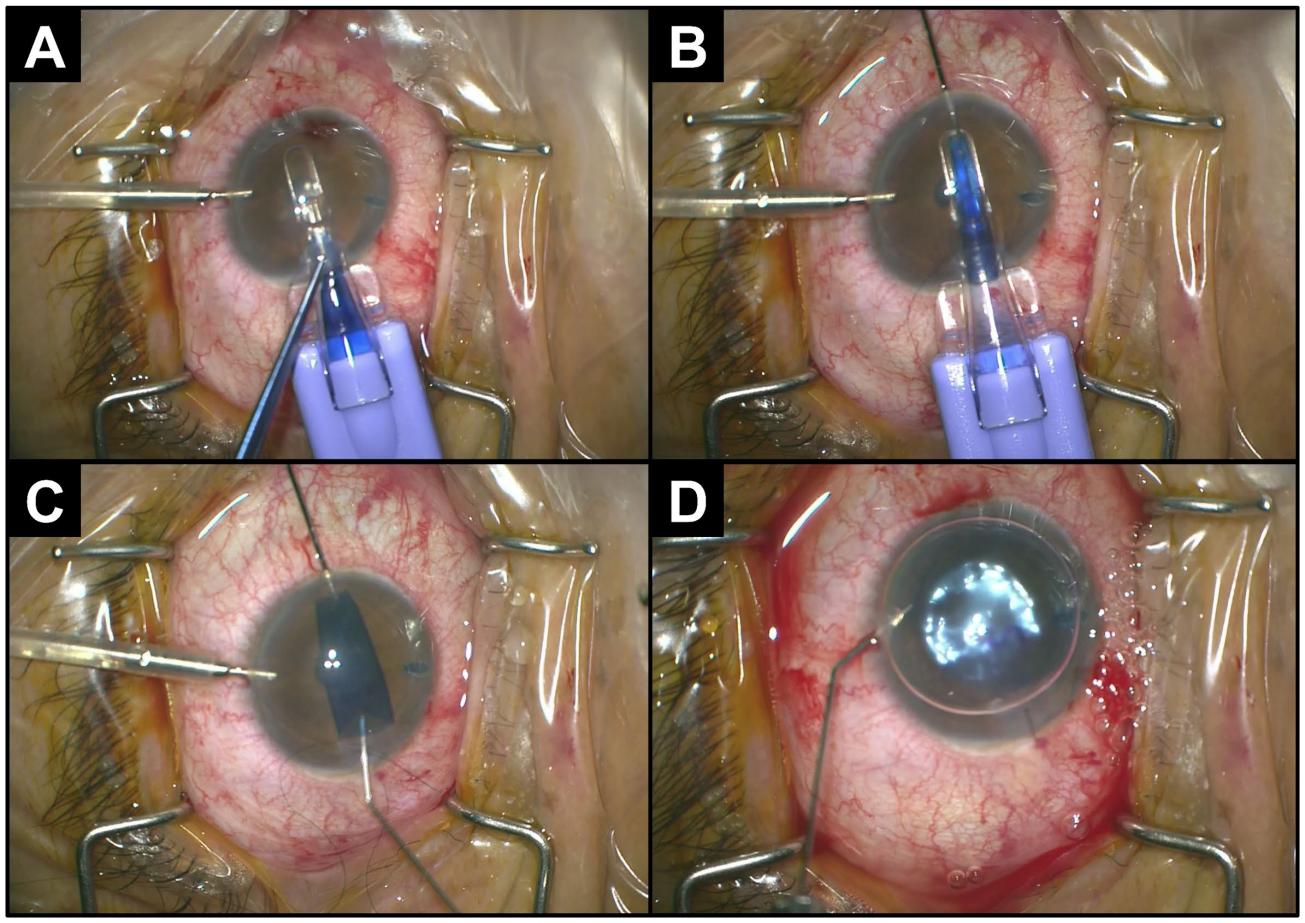
Intra-operative procedure for endothelium-in pull-through-DMEK using a graft-loaded cartridge. **(A)** Insertion of the cartridge into the anterior chamber through a clear cornea incision. **(B)** Grasping and pulling-through of graft with forceps. **(C)** Natural unfolding of the graft with endothelium-down. **(D)** Injection of gas to tamponade the donor graft to the recipient cornea.

In initial earlier DMEKs, donor corneas were prepared intra-operatively, which was time-consuming and required surgical dexterity with a steep learning curve. The concept of a preloaded DMEK was introduced to reduce intra-operative time, costs, and stress with graft preparation (8). This pre-operative preparation involves stripping, trephining, and folding into a defined orientation before loading into a cartridge device for storage and delivery into the recipient anterior chamber (AC) (9). The use of such cartridges with the graft endothelium tri-folded inwards (“endothelium-in”) has been coupled with “pull-through” techniques (Figure 1), where the donor graft is manually grasped and orientated into place, as compared to conventional techniques of graft injection into the AC (“injector-DMEK”) (10–12).

Some studies have reported reduced early complications, such as rebubbling for preloaded DMEKs versus intra-operatively surgeon-loaded DMEKs (13, 14), while others have reported increased rebubbling rates (15). There currently are few direct comparative studies highlighting intra-operative complications and difficulties with preloaded DMEKs. Additionally, there is a lack of literature on the outcomes of preloaded DMEKs following international transportation. Hence, we present here a prospective study of our first 20 imported and preloaded endothelium-in DMEKs, compared with surgeon-loaded pull-through-DMEKs in Asian eyes—with a focus on intra-operative complications, early post-operative complications, visual outcomes, and endothelial cell loss (ECL) within the first 6 months post-operatively.

## Materials and methods

### Study design and participants

We conducted a prospective study of 60 DMEK surgeries that utilized the endothelium-in pull-through technique, completed by a single experienced cornea specialist (MA) at a tertiary ophthalmology center—Singapore National Eye Centre from August 2020 to June 2024. Twenty of these cases were imported preloaded DMEKs, each then matched with two cases of surgeon-loaded DMEKs based on donor age within 3 years. Basic demographic data, clinical outcomes, surgical operation times, best corrected visual acuity (BCVA), endothelial cell density (ECD), and DMEK graft donor details were compiled from electronic health records. Our study was conducted as part of the Singapore Corneal Transplant Registry, which monitors clinical data and outcomes of corneal transplants in Singapore (16), with the approval of the local institutional review board (CIRB Ref 2011/577/A), and in accordance with the Declaration of Helsinki. Written informed consent was obtained from all subjects. Sample size calculation was based on a previous study by Romano et al., reporting rebubbling rates of 48% in preloaded and 15% in surgeon-loaded DMEKs (15). A total sample size of 60 would be required to detect differences in rebubbling rates between preloaded and surgeon-loaded cases at a significance level of 0.05 and a power of 80%.

### Graft preparation

For surgeon-loaded cases, the donor cornea was prepared by the surgeon (MA) intra-operatively using the SCUBA technique (17), followed by trephination according to the required size. The graft was then tri-folded after staining and pulled into the Endoglide cartridge (CORONET DMEK Endoglide; Network Medical Products, United Kingdom). Preloaded grafts were imported internationally as preloaded Endoglide cartridges (Preloaded DMEK Endoglide; Eversight, United States). The technique employed by the eye bank for loading the donor DMEK graft into the device has been previously described (18, 19). The graft processing-to-DMEK durations for all preloaded DMEKs were between 72 and 96 h.

### Surgical procedure

DMEK surgical techniques were generally as previously described (11, 20, 21). A clear cornea incision was made with two side ports. An AC maintainer was placed. Peripheral iridotomy was performed. Recipient descemetorhexis was performed using a reverse Sinskey hook. The Endoglide cartridge was inserted into the AC, and the graft was manually grasped using 27G curved Endoglide forceps from a paracentesis at the opposite side of the cornea and pulled through into the AC. Once the graft was fully unfolded and in place at the center of the host cornea, 20% sulfur hexafluoride gas was injected to achieve 80% fill to tamponade the graft. All wounds were closed with a single 10/0 nylon suture.

### Follow-up and post-operative management

As previously described (21), all patients remained in face-up posture for at least 2 h post-operation and had intraocular pressure (IOP) routinely assessed and managed with topical or medical treatment before discharge. All patients received standard post-operative topical antibiotics (levofloxacin 0.5%; Santen, Japan) and topical corticosteroids (prednisolone acetate; Allergan, United States) following a standard tapering dose regime as previously described (22). Patients generally had follow-up visits at 1 day, 1 month, 3 months, and 6 months post-operatively. At follow-up visits, they were examined by slit-lamp biomicroscopy, and BCVA was measured using the Snellen visual acuity chart in the logarithm of minimum angle of resolution (logMAR) units for analysis (23). Central corneal ECD was measured via non-contact specular microscopy (CellChek 20; Konan Medical Corp, Japan or CEM-530; Nidek, Japan) by certified ophthalmic technicians as previously described (24). ECD values were obtained from the built-in automatic endothelial cell segmentation software using the center method to measure cell area.

### Clinical outcomes

The main clinical outcomes monitored are intra-operative and early post-operative complications up to 6 months post-operatively. Intra-operative complications recorded were as previously defined: (21) donor graft tears, folded graft edges requiring manual unfolding with marginal dissector, excessive bleeding, de-centered graft, incorrect graft orientation (upside-down, “endothelium-up”), high vitreous pressure, unstable AC, graft extrusion from pull-through cartridge, and conversion to a standard injector technique DMEK. Post-operative complications recorded were: cystoid macular edema, early immune-mediated rejection signs, partial detachments (lack of adherence in <30% of the graft surface area) (22), need for rebubbling, corneal edema or haze, and new-onset glaucoma or ocular hypertension.

### Statistical analyses

SPSS 26.0 (IBM Corp, United States) and GraphPad Prism software (Prism; GraphPad, United States) were used for all statistical analyses in this study. Descriptive statistics included mean ± standard deviation for continuous variables, whereas categorical variables included frequency distribution and percentages in parentheses. All between-group comparisons of continuous parameters were performed using independent t-tests (paired for applicable comparisons). All between-group comparisons of categorical parameters were performed using Fisher’s exact or chi-square tests. Linear regression was used for scatter plot estimation and multivariable analysis. Multivariable analysis models were built using potential confounding independent variables that were found to be significantly different from the univariable analysis. Upper and lower 95% confidence intervals (CIs) were used for graphs and means. Tests were two-sided, with statistical significance set at *p* < 0.05. Logistic regression analysis was reported as odds ratio (OR). Statistical significance was indicated with a single asterisk for *p* < 0.05, double asterisks for *p* < 0.01, and triple asterisks for *p* < 0.001. ECL was defined as the percentage loss of ECD compared to the pre-operative donor ECD. At a 0.05 significance level with group sample sizes of 20 and 40, there is 80% statistical power to detect differences between the groups greater or equal to 0.767 of the standard deviation.

## Results

We analyzed 60 eyes from 60 patients who underwent pull-through DMEK at a mean age of 69.1 (Table 1), with 70% (*n* = 42) male patients and 30% (*n* = 18) female patients. The mean donor age was 64.4. Twenty eyes underwent preloaded endothelium-in DMEK and were each case-matched with two cases of surgeon-loaded DMEKs (20 preloaded vs. 40 surgeon-loaded). Overall, 27% (*n* = 16) of eyes had Fuchs’ endothelial cell dystrophy (FECD) and 73% (*n* = 44) had pseudophakic bullous keratopathy (PBK).

**Table 1:** Descriptive characteristics of the patients undergoing preloaded and surgeon-loaded DMEKs.

Characteristics	All eyes *n* = 60	DMEK graft preparation		*P*
		Preloaded	Surgeon-loaded	
		*n* = 20	*n* = 40	
Age at DMEK	69.1 ± 11.0	66.1 ± 12.9	70.6 ± 9.7	0.137
Ethnicity				
Chinese	49 (81.7%)	17 (85.0%)	38 (95.0%)	0.071
Indian	2 (3.3%)	2 (10.0%)	0	
Malay	1 (1.7%)	1 (5.0%)	0	
Others	2 (3.3%)	0	2 (5.0%)	
Gender				
Male	42 (70.0%)	14 (70.0%)	28 (70.0%)	1.00
Female	18 (30.0%)	6 (30.0%)	12 (30.0%)	
Glaucoma*				
Primary open angle	7 (11.7%)	2 (10.0%)	5 (12.5%)	1.00
Primary closed angle	7 (11.7%)	4 (20.0%)	3 (7.5%)	0.208
Secondary glaucoma	4 (6.7%)	1 (5.0%)	3 (7.5%)	1.00
Not glaucomatous	42 (70.0%)	13 (65.0%)	29 (72.5%)	0.550
Indication for DMEK				
FECD	16 (26.7%)	8 (40.0%)	8 (20.0%)	0.099
PBK^†^	44 (73.3%)	12 (60.0%)	32 (80.0%)	
Donor graft characteristics				
Donor age	64.4 ± 7.1	64.2 ± 7.6	64.6 ± 7.0	0.860
Donor ECD^†^ (cells/mm^2^)	2800 ± 223	2766 ± 174	2818 ± 246	0.404
Donor graft diameter^†^ (mm)	7.82 ± 0.25	7.80 ± 0.25	7.83 ± 0.25	0.666

FECD: Fuchs’ endothelial cell dystrophy, PBK: pseudophakic bullous keratopathy, ECD: endothelial cell density.*Only one patient had a trabeculectomy, for primary open angle glaucoma, belonging to the preloaded group.^†^Iridocorneal endothelial syndrome and previous graft failures requiring regrafts listed as PBK indication.

### Intra-operative findings

Table 2 summarizes the main clinical outcomes for both techniques. Overall, preloaded DMEKs were significantly shorter in duration than surgeon-loaded DMEKs (26.2 min vs. 39.5 min; *p* < 0.001). Preloaded DMEK was associated with an increased conversion rate to the standard DMEK technique of injector-DMEK (15% vs. 0%; *p* = 0.033). However, there were no significant differences in the overall rate of intra-operative complications (40% vs. 22.5%; *p* = 0.156). From the start of the operation, 10% (*n* = 2) of the preloaded grafts were incorrectly oriented in the cartridge (endothelium-out instead of endothelium-in), while none of the surgeon-loaded cartridges were incorrectly oriented. Similarly, 10% (*n* = 2) of the preloaded grafts instantly extruded when inserted into the AC, resulting in a conversion to injector-DMEK.

**Table 2:** Comparison of intra-operative complications, early post-operative complications, final graft outcomes, endothelial cell density, and visual outcomes between preloaded and surgeon-loaded DMEKs.

Characteristics	All eyes *n* = 60	DMEK graft preparation		*P*
		Preloaded	Surgeon-loaded	
		*n* = 20	*n* = 40	
Intra-operative time^†^ (min)	34.8 ± 14.6	26.2 ± 8.8	39.5 ± 15.1	<0.001**
Intra-operative complications				
Any complication	17 (36.3%)	8 (40.0%)	9 (22.5%)	0.156
Donor graft tear	3 (5.0%)	1 (5.0%)	2 (5.0%)	1.00
Folded graft edge	5 (8.3%)	2 (10.0%)	3 (7.5%)	1.00
Bleeding	2 (3.3%)	0	2 (5.0%)	0.548
De-centered graft	1 (1.7%)	0	1 (2.5%)	1.00
Upside-down graft	2 (3.3%)	2 (10.0%)	0	0.107
High vitreous pressure	3 (5.0%)	2 (10.0%)	1 (2.5%)	0.255
Graft extrusion from cartridge	2 (3.3%)	2 (10.0%)	0	0.107
Converted to injector-DMEK	3 (5.0%)	3 (15.0%)	0	0.033*
Early post-operative complications				
Any complication	19 (31.7%)	7 (35.0%)	12 (30.0%)	0.772
Cystoid macula oedema	1 (1.7%)	1 (5.0%)	0	0.333
Early rejection signs	2 (3.3%)	0	2 (5.0%)	0.548
Partial graft detachment	5 (8.3%)	2 (10.0%)	3 (7.5%)	1.00
Rebubbling required	3 (5.0%)	1 (5.0%)	2 (5.0%)	1.00
Corneal haze/oedema	5 (8.3%)	2 (10.0%)	3 (7.5%)	1.00
Ocular hypertension	10 (16.7%)	3 (15.0%)	7 (17.5%)	1.00
Final graft outcome				
Clear, functional & surviving	59 (98.3%)	20 (100%)	39 (97.5%)	1.00
Graft failure	1 (1.7%)	0	1 (2.5%)	
Endothelial cell density				
Donor ECD (cells/mm^2^)	2,797 ± 221	2,766 ± 174	2,812 ± 242	0.450
1 M post-DMEK ECD^‡^	1778 ± 652	1,641 ± 733	1941 ± 528	0.269
3 M post-DMEK ECD^§^	1770 ± 555	1862 ± 458	1715 ± 612	0.519
6 M post-DMEK ECD^¶^	1,659 ± 499	1,535 ± 553	1715 ± 475	0.329
Visual acuity (logMAR)				
Pre-DMEK BCVA	1.08 ± 0.73	1.05 ± 0.76	1.09 ± 0.72	0.843
1 M post-DMEK BCVA	0.51 ± 0.42	0.57 ± 0.49	0.48 ± 0.39	0.443
3 M post-DMEK BCVA^#^	0.48 ± 0.48	0.48 ± 0.49	0.48 ± 0.48	1.00
6 M post-DMEK BCVA^#^	0.57 ± 0.62	0.66 ± 0.74	0.53 ± 0.55	0.455
% BCVA improvement^††^	58.4 ± 44.5	60.3 ± 44.4	57.4 ± 45.1	0.816
BCVA at 6/12 or better^††^	35 (58.3%)	12 (60.0%)	23 (57.5%)	1.00

logMAR: logarithm of minimum angle of resolution, ECD: endothelial cell density, ECL: endothelial cell loss, BCVA: best corrected visual acuity.^*^Bolded P-values with asterisks indicate statistical significance.^†^Number of patients with intra-operative time data: 20 preloaded, 36 surgeon-loaded, total 56.^‡^ Number of patients with 1-month post-DMEK ECD data: 13 preloaded, 11 surgeon-loaded, total 24. ^§^ Number of patients with 3-month post-DMEK ECD data: 10 preloaded, 17 surgeon-loaded, total 27. ^¶^ Number of patients with 6-month post-DMEK ECD data: 11 preloaded, 24 surgeon-loaded, total 35. ^#^ Number of patients with 3-month and 6-month post-DMEK BCVA data: 19 preloaded, 39 surgeon-loaded, total 59. ^††^ Best BCVA score within 6-months post-DMEK used for % improvement and achievement of 6/12 BCVA.

### Early post-operative complications

Overall, there were no significant differences in early post-operative complication rates in preloaded versus surgeon-loaded DMEKs (35% vs. 30%; *p* = 0.772). Post-operative partial detachment of the graft was not significantly different between preloaded versus surgeon-loaded DMEKs (10% vs. 7.5%; *p* > 0.999). Of the five cases of partial detachment, only one preloaded DMEK case (5%) required rebubbling, while two surgeon-loaded cases (5%) required rebubbling. There were no significant differences in rebubbling rates between preloaded and surgeon-loaded DMEKs (5% vs. 5%; *p* > 0.999). Overall, all cases of partial detachment either self-resolved or were treated by rebubbling without any further complications. All 10 cases of post-operative ocular hypertension were steroid-responsive IOP elevations that were resolved with topical glaucoma drugs, with none resulting in glaucomatous damage. Only one case in this study ultimately had graft failure at 6 months due to intraocular lens-induced pigment dispersion, inflammation, and endothelial cell loss, belonging to the surgeon-loaded group. Multivariable logistic regression was employed to investigate variables that increased the risk of intra- or post-operative complications (Table 3). Preloaded DMEK, having PBK and lower donor ECD, had increased ORs, but overall, no variables were found to be statistically significant. Preloaded DMEK had an OR of 2.69 for intra-operative complications (*p* = 0.156) and 1.75 for early post-operative complications (*p* = 0.371) compared to surgeon-loaded DMEK. Preloaded DMEK was not associated with increased rates of any complications after adjusting for confounders (OR = 2.04; *p* = 0.371).

**Table 3:** Multivariable logistic regression of any intra-operative and early post-operative complications.

Variables	Any intra-operative complications		Any early post-operative complications		Any intra- or early post-operative complications	
	Odds ratio (95% CIs)	*P*	Odds ratio (95% CIs)	*P*	Odds ratio (95% CIs)	*P*
Preloaded DMEK (vs. surgeon-loaded)	2.69 (0.692–11.2)	0.156	1.75 (0.511–6.04)	0.371	2.04 (0.633–7.02)	0.242
Male gender	0.374 (0.0703–1.81)	0.228	1.05 (0.244–4.73)	0.943	0.772 (0.192–3.08)	0.713
DMEK recipient age	0.984 (0.915–1.05)	0.644	0.99 (0.932–1.05)	0.743	0.989 (0.931–1.05)	0.728
Glaucoma	0.281 (0.0439–1.34)	0.136	0.873 (0.221–3.22)	0.841	0.44 (0.114–1.57)	0.215
DMEK indication of PBK (vs. FECD)	1.39 (0.220–9.76)	0.728	1.88 (0.347–11.6)	0.472	1.57 (0.311–8.41)	0.586
Donor ECD (per 100 cells/mm^2^)	0.792 (0.532–1.10)	0.206	0.941 (0.679–1.26)	0.690	0.887 (0.657–1.16)	0.398
Donor graft diameter (per mm)	11.7 (0.709–333)	0.112	0.738 (0.071–8.26)	0.798	1.84 (0.186–19.5)	0.600

FECD: Fuchs’ endothelial cell dystrophy, PBK: pseudophakic bullous keratopathy, ECD: endothelial cell density.

### Post-operative endothelial cell density and loss

**Figure 2:**
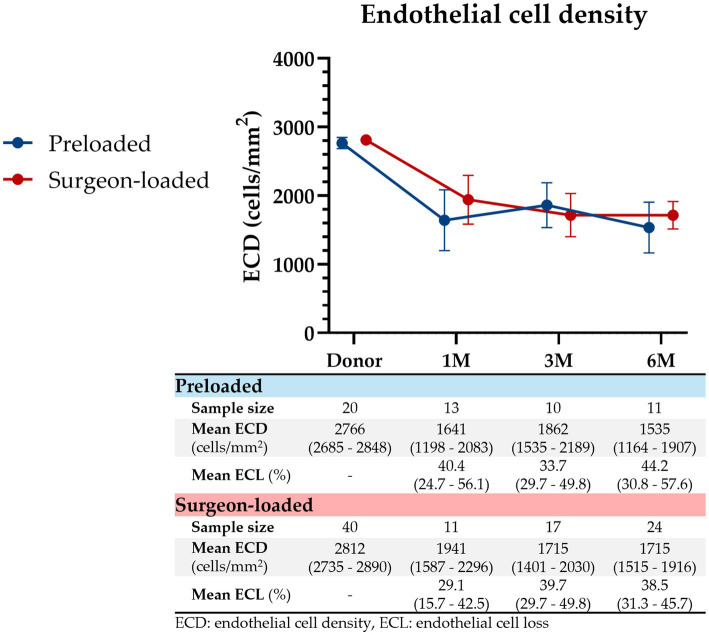
Endothelial cell density post-DMEK. ECD represented from pre-DMEK (donor ECD) to 1 month, 3 months, and 6 months post-DMEK for preloaded (blue) and surgeon-loaded (red) DMEKs. ECL, as a percentage of the pre-operative donor ECD, is shown below as well. Vertical error bars represent the 95% CIs. At all four timepoints, there were no significant differences in ECD (*p* = 0.450, 0.269, 0.519, 0.329, respectively) or ECL (*p* = 0.251, 0.402, 0.392, respectively).

We detected no difference in ECD (Figure 2) between preloaded and surgeon-loaded DMEKs at 1 month (1641 vs. 1941 cells/mm^2^; *p* = 0.269), 3 months (1862 vs. 1715 cells/mm^2^; *p* = 0.519), and 6 months post-operatively (1535 vs. 1715 cells/mm^2^; *p* = 0.329). ECL, as a percentage of the pre-operative donor ECD, was not found to be significantly different between preloaded and surgeon-loaded DMEKs at 1 month (40.4% vs. 29.1%; *p* = 0.251), 3 months (33.7% vs. 39.7%; *p* = 0.402), and 6 months post-operatively (44.2% vs. 38.5%; *p* = 0.392).

### Visual outcomes

**Figure 3:**
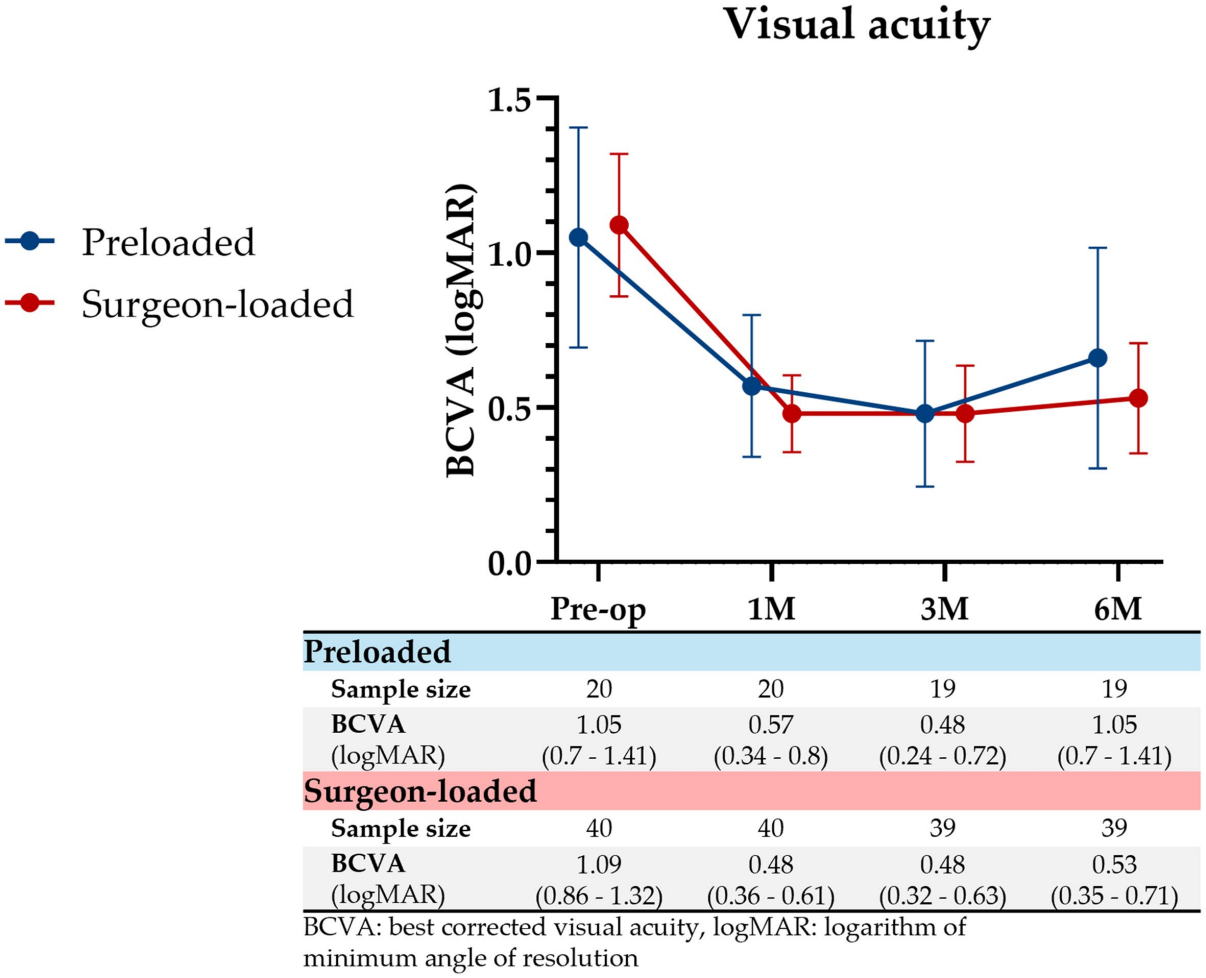
Visual acuity post-DMEK. BCVA from pre-DMEK to 1 month, 3 months, and 6 months post-DMEK, for preloaded (blue) and surgeon-loaded (red) DMEKs. Vertical error bars represent the 95% CIs. There were no significant differences in BCVA at all four timepoints (*p* = 0.843, 0.443, 1.00, 0.455, respectively).

Pre-operative BCVA was similar for preloaded and surgeon-loaded groups (logMAR 1.05 vs. 1.09, *p* = 0.843). Visual acuity (Figure 3) was similar for both groups at 1 month (logMAR 0.57 vs. 0.48, *p* = 0.443), 3 months (logMAR 0.48 vs. 0.48, *p* > 0.999), and 6 months post-operatively (logMAR 0.66 vs. 0.53, *p* = 0.455). Improvements in BCVA were similar (60.3% vs. 57.4%; *p* = 0.816). 60% (*n* = 12) of preloaded and 57.5% (*n* = 23) of surgeon-loaded DMEKs achieved BCVA of 6/12 or better (*p* > 0.999).

## Discussion

In our direct comparative analysis of preloaded versus surgeon-loaded DMEKs performed in Asian eyes, we found that both techniques had similar clinical outcomes in terms of complications, visual outcomes, and ECL. There were no significant differences in overall rates of intra- or early post-operative complications in preloaded versus surgeon-loaded DMEKs in the univariable and multivariable analyses. These findings demonstrate that preloaded DMEK is overall safe and comparable in outcomes to surgeon-loaded DMEK, when the endothelium-in bimanual pull-through technique is utilized.

Current studies for endothelium-in pull-through-DMEK report reduced intra-operative endothelial cell losses (12, 25), with similar clinical outcomes to endothelium-out injector techniques (26–28). From the context of practicality, compared to injector-DMEK, where the donor graft freely floats in the AC and surgical dexterity is critical (29), pull-through techniques allow direct control of the donor graft, reducing surgical unpredictability (30). This is especially important in eyes with difficult visualization and shallow ACs (31), such as Asian eyes with smaller, deeper-set eyes, higher vitreous pressures, and thick brown irises (11, 28, 32). Furthermore, in the context of preloading, the endothelium-in storage and transportation of preloaded grafts have demonstrated slight improvements in cell viability versus preloaded endothelium-out grafts (33). That being said, endothelium-out grafts preloaded into injectors are more common and have been around for longer (34), and have also similarly shown comparable clinical outcomes to the traditional surgeon-prepared endothelium-out injector-DMEKs (35).

In our study, preloaded DMEK resulted in significantly saved intra-operative time (26.2 min vs. 39.5 min; *p* < 0.001). This is one of the many factors that can translate to reduced costs for society, healthcare, and the patient. Böhm et al. demonstrated greater cost savings and incremental cost–utility ratio with preloaded DMEKs, generating slightly greater utility in terms of quality-adjusted life years values relative to surgeon-loaded DMEKs (36). There is also an important clinical benefit of mitigating intra-operative stress (37), minimizing the risk of tissue wastage, cancellation, and postponement of DMEK due to failure of intra-operative graft preparation (38–40). On the other hand, accurate pre-operative selection of graft size is a key requirement in preloaded DMEKs, as they cannot be trephined again intra-operatively. Endothelial cell losses during storage have been highlighted as another potential concern; however, studies have shown that storage for up to 48 h does not negatively affect graft attachment or endothelial cell survival compared to freshly prepared grafts (40–42). Nonetheless, it has been found that endothelial cell viability significantly declines 4 days after eye bank processing, suggesting that the processing-to-DMEK duration should not exceed 96 h (43). Currently, ECD measurement of preloaded grafts is only possible through Straiko Modified Jones tubes (44), but not cartridges. Future developments in ECD assessment of the donor graft while in the preloaded cartridge would be beneficial for donor tissue quality assurance and patient outcomes, especially when graft tissues are internationally imported and have storage times longer than 48 h. Some other external factors should also be considered in the international supply chain of cornea tissues: transnational legislation, eye bank pricing, and unexpected delays or disturbances to the supply chain. In Singapore, the local supply of donated cornea tissue is low, resulting in our dependence on internationally ordered and imported cornea tissue.

Another concern with preloaded DMEK is the early post-operative complications of graft detachment and consequent rebubbling procedures (43, 45). In our study, post-operative graft detachment was 10% in the preloaded group versus 7.5% in the surgeon-loaded group; however, this difference was not statistically significant (*p* > 0.999). All cases of graft detachment in our study were resolved over time without further complications, with or without rebubbling. The rebubbling rate was 5% for both preloaded and surgeon-loaded groups. In their clinical and laboratory study, Romano et al. demonstrated greater detachment and rebubbling rates in preloaded versus surgeon-loaded grafts, suggesting that preloaded grafts might be less stiff and adhesive due to time in storage, possibly increasing the risk of detachment (15). Conversely, Böhm et al. demonstrated the opposite conclusion—lower rebubbling rate for preloaded DMEK in their study involving cornea fellows starting on the learning curve, suggesting that less experienced surgeons might face more complications in traditional DMEKs due to the additional component of intra-operative graft preparation (14). The use of preloaded DMEK might serve as a stepping stone for the training of newer cornea surgeons, as the task of intra-operative graft preparation is accomplished by the eye bank. These two studies demonstrate that the rebubbling rate in preloaded DMEKs is multi-factorial and is also likely to be center- and surgeon-dependent, especially given that it is a decision based on clinical criteria (46). Our study is similar to the works of Potts et al. and Cho et al., demonstrating that preloaded and surgeon-loaded DMEKs have no significant differences in rebubbling rate (35, 47). Rebubbling is an important variable in slowed visual recovery and increased endothelial cell losses post-DMEK (48), and we have demonstrated comparable rebubbling rates and clinical outcomes for preloaded DMEK.

Given that the 20 preloaded cases here represent the surgeon’s first 20 preloaded DMEK cases, there were some difficulties encountered worth highlighting for the benefit of other surgeons considering preloaded DMEKs. Within our preloaded group, 15% (*n* = 3) of the DMEKs had to be converted intra-operatively to the standard injector-DMEK technique. We found that pre-operative preparation for potential conversion to injector-DMEK was important. Specifically, the availability of surgical equipment and the preparedness of the surgical staff were factors that should be considered when using preloaded grafts. In two of our three conversion cases, the main reason was the extrusion of the grafts from the device into the AC immediately upon cartridge insertion before the surgeon could grasp the grafts with forceps. This could be due to several reasons, namely, unstable AC-cartridge fluid dynamics, the type of solution loaded in the cartridge, or possibly the loading process of the graft into the cartridge at the eye bank. In our experience with surgeon-loaded pull-through-DMEKs, we did not previously encounter any form of uncontrolled graft extrusion from the cartridge. To the best of our knowledge, there has not been any literature citing this intra-operative complication from preloaded DMEK. We also encountered two occasions (10%) where the graft was loaded upside-down in the cartridge pre-operatively. They were tri-folded with the endothelium inwards but were “endothelium-up” once inserted into the AC. The incorrect orientations were only observable once unfolded in the AC. Intra-operative maneuvers to flip the grafts were successful in both cases; however, this negated the benefit of bimanual control of the graft. Younger donor grafts, in general, tend to be tighter and smaller in conformation, possibly allowing for spontaneous rotations during storage, leading to upside-down grafts (49, 50). In both cases we encountered, the donor age was 55 and 61 years, below the mean preloaded donor age of 64 years. Overall, circumvention of incorrect graft orientation requires surgeon experience in recognizing misoriented grafts, surgical dexterity, and graft marking with an “F,” “S,” “I-II,” or any other asymmetric stamp (51–53). In this study, “S” stamps were ordered for the preloaded grafts and self-marked in the surgeon-loaded DMEK cases. Slit-beam light sources or anterior segment optical coherence tomography (AS-OCT) are other possible adjuncts for determining correct graft orientation intra-operatively (54).

The study we present here on preloaded DMEK in Asian eyes seeks to contribute to the growing body of knowledge regarding preloaded DMEKs. A key strength of this study is the novelty of international transportation of preloaded endothelium-in DMEK grafts for a total travel time greater than 72 h. At the same time, we report that our preloaded DMEKs had comparable clinical outcomes as surgeon-loaded cases; future randomized controlled trials might be required to investigate this conclusively. We could not do so in this study, given that this study represents our first 20 preloaded cases, and randomizing would not have been logistically feasible.

Despite 58.3% (*n* = 35) of patients achieving visual acuity at 6/12 or better (logMAR 0.3), the mean post-operative visual acuity appears to be poor at logMAR 0.57. This is likely because there are a few isolated cases with clear and functioning corneas but poor vision due to co-morbid uncontrolled glaucoma and/or other retinal pathologies. These few cases with visual acuity worse than 6/60 (logMAR 1.0) might have contributed to the seemingly poor post-operative visual acuity despite the majority of patients achieving acceptable, if not good, vision post-DMEK. While they might slightly confound visual outcomes, they were nonetheless not excluded as this better represents the typical clinical scenarios encountered in our clinical context.

We recognize that DMEK indications and outcomes could possibly differ from Asian to a global population and that our sample size might be limited for generalized application. Nonetheless, in our experience, the preloaded endothelium-in DMEK is a valuable technique and option for cornea surgeons to provide surgical predictability and shorter operation times. In conclusion, we report and directly compared endothelium-in preloaded and surgeon-loaded DMEKs, demonstrating similar clinical outcomes with significantly increased intra-operative time savings. Given the challenges and costs of intra-operative graft preparation, preloading might be an exciting prospect for the future of DMEKs.

## Data Availability

The original contributions presented in the study are included in the article/supplementary material, further inquiries can be directed to the corresponding author.

## References

[1] Joyce NC. Proliferative capacity of the corneal endothelium. Prog Retin Eye Res. (2003) 22:359–89. doi: 10.1016/s1350-9462(02)00065-4, PMID: 12852491

[2] Bourne WM. Biology of the corneal endothelium in health and disease. Eye (Lond). (2003) 17:912–8. doi: 10.1038/sj.eye.6700559, PMID: 14631396

[3] Gain P Jullienne R He Z Aldossary M Acquart S Cognasse F. Global survey of corneal transplantation and eye banking. JAMA Ophthalmol. (2016) 134:167–73. doi: 10.1001/jamaophthalmol.2015.4776, PMID: 26633035

[4] Melles GR Ong TS Ververs B van der Wees J. Descemet membrane endothelial keratoplasty (DMEK). Cornea. (2006) 26:199–206. doi: 10.1097/01.ico.0000248385.16896.34, PMID: 17102683

[5] Price FW Jr Price MO. Evolution of endothelial keratoplasty. Cornea. (2013) 32:S28–32. doi: 10.1097/ICO.0b013e3182a0a307, PMID: 24104929

[6] Santander-García D Dapena I Baydoun L Melles GRJ. DMEK complications: current treatment and recommendations. Expert Rev Ophthal. (2018) 13:33–46. doi: 10.1080/17469899.2018.1429917

[7] Moura-Coelho N Papa-Vettorazzi R Reyes A Cunha JP Guell JL. Ultrathin DSAEK versus DMEK—review of systematic reviews. Eur J Ophthalmol. (2023) 34:913–23. doi: 10.1177/11206721231214605, PMID: 37964555

[8] Parekh M Ruzza A Ferrari S Busin M Ponzin D. Preloaded tissues for Descemet membrane endothelial Keratoplasty. Am J Ophthalmol. (2016) 166:120–5. doi: 10.1016/j.ajo.2016.03.048, PMID: 27066719

[9] Busin M Leon P D'Angelo S Ruzza A Ferrari S Ponzin D. Clinical outcomes of preloaded Descemet membrane endothelial Keratoplasty grafts with endothelium tri-folded inwards. Am J Ophthalmol. (2018) 193:106–13. doi: 10.1016/j.ajo.2018.06.013, PMID: 29940169

[10] Busin M Leon P Scorcia V Ponzin D. Contact Lens-assisted pull-through technique for delivery of tri-folded (endothelium in) DMEK grafts minimizes surgical time and cell loss. Ophthalmology. (2016) 123:476–83. doi: 10.1016/j.ophtha.2015.10.050, PMID: 26686969

[11] Ang M Mehta JS Newman SD Han SB Chai J Tan D. Descemet membrane endothelial Keratoplasty: preliminary results of a donor insertion pull-through technique using a donor mat device. Am J Ophthalmol. (2016) 171:27–34. doi: 10.1016/j.ajo.2016.08.023, PMID: 27565226

[12] Ong HS Htoon HM Ang M Mehta JS. "endothelium-out" and "endothelium-in" Descemet membrane endothelial Keratoplasty (DMEK) graft insertion techniques: a systematic review with Meta-analysis. Front Med (Lausanne). (2022) 9:868533. doi: 10.3389/fmed.2022.868533, PMID: 35775001 PMC9237218

[13] Juratli L Qureshi S Liles N Hussain M Hood C Mian SI. Clinical outcomes of prestripped, prestained, and preloaded Descemet's membrane endothelial keratoplasty ("P3 DMEK"). Taiwan J Ophthalmol. (2023) 13:55–61. doi: 10.4103/tjo.TJO-D-22-00155, PMID: 37252165 PMC10220431

[14] Bohm MS Wylegala A Leon P Ong Tone S Ciolino JB Jurkunas UV. One-year clinical outcomes of preloaded Descemet membrane endothelial Keratoplasty versus non-preloaded Descemet membrane endothelial Keratoplasty. Cornea. (2021) 40:311–9. doi: 10.1097/ICO.0000000000002430, PMID: 32740011

[15] Romano V Kazaili A Pagano L Gadhvi KA Titley M Steger B. Eye bank versus surgeon prepared DMEK tissues: influence on adhesion and re-bubbling rate. Br J Ophthalmol. (2022) 106:177–83. doi: 10.1136/bjophthalmol-2020-317608, PMID: 33127828 PMC8788033

[16] Tan D Ang M Arundhati A Khor WB. Development of selective lamellar Keratoplasty within an Asian corneal transplant program: the Singapore corneal transplant study (an American ophthalmological society thesis). Trans Am Ophthalmol Soc. (2015) 113:T10. PMID: 26755854 PMC4692329

[17] Price MO Giebel AW Fairchild KM Price FW Jr. Descemet's membrane endothelial keratoplasty: prospective multicenter study of visual and refractive outcomes and endothelial survival. Ophthalmology. (2009) 116:2361–8. doi: 10.1016/j.ophtha.2009.07.010, PMID: 19875170

[18] Kim M Kim KH Lee HK. Clinical outcomes of Descemet membrane endothelial Keratoplasty using a preloaded imported graft. Korean J Ophthalmol. (2023) 37:373–9. doi: 10.3341/kjo.2023.0053, PMID: 37562438 PMC10587462

[19] Hooton J Kim KH Lentz SI Hicks N Jones K McCoy K. Safety of long-term storage and shipping of Prestripped, Prestained, and preloaded Descemet membrane endothelial Keratoplasty tissue. Cornea. (2019) 38:1023–8. doi: 10.1097/ICO.0000000000001974, PMID: 31090594 PMC7315383

[20] Ang M Wilkins MR Mehta JS Tan D. Descemet membrane endothelial keratoplasty. Br J Ophthalmol. (2016) 100:15–21. doi: 10.1136/bjophthalmol-2015-306837, PMID: 25990654

[21] Ang M Ting DSJ Kumar A May KO Htoon HM Mehta JS. Descemet membrane endothelial Keratoplasty in Asian eyes: intraoperative and postoperative complications. Cornea. (2020) 39:940–5. doi: 10.1097/ICO.0000000000002302, PMID: 32452991

[22] Woo JH Ang M Htoon HM Tan D. Descemet membrane endothelial Keratoplasty versus Descemet stripping automated endothelial Keratoplasty and penetrating Keratoplasty. Am J Ophthalmol. (2019) 207:288–303. doi: 10.1016/j.ajo.2019.06.012, PMID: 31228467

[23] Bailey IL Lovie JE. New design principles for visual acuity letter charts. Am J Optom Physiol Optic. (1976) 53:740–5. doi: 10.1097/00006324-197611000-00006, PMID: 998716

[24] Ang M Mehta JS Anshu A Wong HK Htoon HM Tan D. Endothelial cell counts after Descemet's stripping automated endothelial keratoplasty versus penetrating keratoplasty in Asian eyes. Clin Ophthalmol. (2012) 6:537–44. doi: 10.2147/OPTH.S26343, PMID: 22536049 PMC3334224

[25] Chong EW Bandeira F Finn P Mehta JS Chan E. Evaluation of Total donor endothelial viability after endothelium-inward versus endothelium-outward loading and insertion in Descemet membrane endothelial Keratoplasty. Cornea. (2020) 39:104–9. doi: 10.1097/ICO.0000000000002014, PMID: 31169609

[26] Parekh M Romano V Hassanin K Testa V Wongvisavavit R Ferrari S. Delivering endothelial Keratoplasty grafts: modern day transplant devices. Curr Eye Res. (2022) 47:493–504. doi: 10.1080/02713683.2021.2016852, PMID: 34986709

[27] Price MO Lisek M Kelley M Feng MT Price FW Jr. Endothelium-in versus endothelium-out insertion with Descemet membrane endothelial Keratoplasty. Cornea. (2018) 37:1098–101. doi: 10.1097/ICO.0000000000001650, PMID: 29863544

[28] Cheong EZK Ng Yin Ling C Wong QY Chua CSQ Htoon HM Ang M. Clinical outcomes of DMEK comparing endothelium-out injector and endothelium-in pull-through techniques in Asian eyes. Front Med. (2025) 12:1555620. doi: 10.3389/fmed.2025.1555620, PMID: 40248087 PMC12003366

[29] Maier AK Gundlach E Schroeter J Klamann MK Gonnermann J Riechardt AI. Influence of the difficulty of graft unfolding and attachment on the outcome in Descemet membrane endothelial keratoplasty. Graefes Arch Clin Exp Ophthalmol. (2015) 253:895–900. doi: 10.1007/s00417-015-2939-9, PMID: 25631845

[30] Viola P Neri E Testa V Parekh M Cian R Grassetto A. Clinical outcomes of preloaded Descemet membrane endothelial Keratoplasty with endothelium inward: a 24-month comparative analysis between Fuchs endothelial corneal dystrophy and bullous keratopathy. Cornea. (2023) 42:1133–9. doi: 10.1097/ICO.0000000000003138, PMID: 36538420

[31] Siggel R Heindl LM Cursiefen C. Descemet membrane endothelial keratoplasty (DMEK) in phakic eyes with shallow anterior chamber. Graefes Arch Clin Exp Ophthalmol. (2015) 253:817–9. doi: 10.1007/s00417-014-2850-9, PMID: 25408423

[32] Hayashi T Oyakawa I Kato N. Techniques for learning Descemet membrane endothelial Keratoplasty for eyes of Asian patients with shallow anterior chamber. Cornea. (2017) 36:390–3. doi: 10.1097/ICO.0000000000001093, PMID: 28002113 PMC5291281

[33] Romano V Parekh M Ruzza A Willoughby CE Ferrari S Ponzin D. Comparison of preservation and transportation protocols for preloaded Descemet membrane endothelial keratoplasty. Br J Ophthalmol. (2018) 102:549–55. doi: 10.1136/bjophthalmol-2017-310906, PMID: 29133296 PMC5890643

[34] Wojcik G Parekh M Romano V Ferrari S Ruzza A Ahmad S. Preloaded Descemet membrane endothelial Keratoplasty grafts with endothelium outward: a cross-country validation study of the DMEK rapid device. Cornea. (2021) 40:484–90. doi: 10.1097/ICO.0000000000002493, PMID: 32947407

[35] Potts LB Bauer AJ Xu DN Chen SY Alqudah AA Sanchez PJ. The last 200 surgeon-loaded Descemet membrane endothelial Keratoplasty tissue versus the first 200 preloaded Descemet membrane endothelial Keratoplasty tissue. Cornea. (2020) 39:1261–6. doi: 10.1097/ICO.0000000000002400, PMID: 32541187

[36] Bohm M Leon P Wylegala A Ong Tone S Condron T Jurkunas U. Cost-effectiveness analysis of preloaded versus non-preloaded Descemet membrane endothelial keratoplasty for the treatment of Fuchs endothelial corneal dystrophy in an academic Centre. Br J Ophthalmol. (2022) 106:914–22. doi: 10.1136/bjophthalmol-2020-317536, PMID: 33637619

[37] Martin AI Devasahayam R Hodge C Cooper S Sutton GL. Analysis of the learning curve for pre-cut corneal specimens in preparation for lamellar transplantation: a prospective, single-Centre, consecutive case series prepared at the lions New South Wales eye Bank. Clin Experiment Ophthalmol. (2017) 45:689–94. doi: 10.1111/ceo.12941, PMID: 28263034

[38] Godinho JV Mian SI. Update on Descemet membrane endothelial keratoplasty. Curr Opin Ophthalmol. (2019) 30:271–4. doi: 10.1097/ICU.0000000000000577, PMID: 31045882

[39] Romano V Pagano L Gadhvi KA Coco G Titley M Fenech MT. Clinical outcomes of pre-loaded ultra-thin DSAEK and pre-loaded DMEK. BMJ Open Ophthalmol. (2020) 5:e000546. doi: 10.1136/bmjophth-2020-000546, PMID: 33094167 PMC7569929

[40] Deng SX Sanchez PJ Chen L. Clinical outcomes of Descemet membrane endothelial keratoplasty using eye bank-prepared tissues. Am J Ophthalmol. (2015) 159:590–6. doi: 10.1016/j.ajo.2014.12.007, PMID: 25526945

[41] Feng MT Burkhart ZN Price FW Jr Price MO. Effect of donor preparation-to-use times on Descemet membrane endothelial keratoplasty outcomes. Cornea. (2013) 32:1080–2. doi: 10.1097/ICO.0b013e318292a7e5, PMID: 23635858

[42] Rickmann A Wahl S Hofmann N Knakowski J Haus A Börgel M. Comparison of preloaded grafts for Descemet membrane endothelial keratoplasty (DMEK) in a novel preloaded transport cartridge compared to conventional precut grafts. Cell Tissue Bank. (2020) 21:205–13. doi: 10.1007/s10561-020-09814-7, PMID: 32016617

[43] Parekh M Pedrotti E Viola P Leon P Neri E Bosio L. Factors affecting the success rate of preloaded Descemet membrane endothelial Keratoplasty with endothelium-inward technique: a multicenter clinical study. Am J Ophthalmol. (2022) 241:272–81. doi: 10.1016/j.ajo.2022.03.009, PMID: 35288072

[44] Tran KD Dye PK Odell K Galloway J Stoeger CG Straiko MD. Evaluation and quality assessment of Prestripped, preloaded Descemet membrane endothelial Keratoplasty grafts. Cornea. (2017) 36:484–90. doi: 10.1097/ICO.0000000000001150, PMID: 28129302

[45] Parekh M Leon P Ruzza A Borroni D Ferrari S Ponzin D. Graft detachment and rebubbling rate in Descemet membrane endothelial keratoplasty. Surv Ophthalmol. (2018) 63:245–50. doi: 10.1016/j.survophthal.2017.07.003, PMID: 28739402

[46] Tapley JL Hill JR Bauer AJ Straiko MMW Straiko MD Terry MA. Rate of endothelial cell loss and graft survival in Descemet membrane endothelial Keratoplasty in eyes requiring a Rebubble. Cornea. (2023) 42:934–9. doi: 10.1097/ICO.0000000000003118, PMID: 36731078

[47] Cho K Ali M Heckenlaible NJ Jabbour S Jun AS Srikumaran D. Outcomes and early complications using an endothelium-in pull-through Descemet membrane endothelial Keratoplasty technique with preloaded versus surgeon-loaded donor tissue in Fuchs patients. Cornea. (2024) 43:591–7. doi: 10.1097/ICO.0000000000003371, PMID: 37607293

[48] Fu L Hollick EJ. Rebubbling and graft detachment in Descemet membrane endothelial keratoplasty using a standardised protocol. Eye (Lond). (2023) 37:2494–8. doi: 10.1038/s41433-022-02362-2, PMID: 36522529 PMC10397279

[49] Heinzelmann S Huther S Bohringer D Eberwein P Reinhard T Maier P. Influence of donor characteristics on descemet membrane endothelial keratoplasty. Cornea. (2014) 33:644–8. doi: 10.1097/ICO.0000000000000106, PMID: 24675376

[50] Chiang E Chen C Barnes K Chaurasia A Rosen A Solar S. A device for preloaded, Trifolded grafts to facilitate Descemet membrane endothelial keratoplasty. J Med Dev. (2019) 13:1932–6181. doi: 10.1115/1.4043739

[51] Wasielica-Poslednik J Schuster AK Rauch L Glaner J Musayeva A Riedl JC. How to avoid an upside-down orientation of the graft during Descemet membrane endothelial Keratoplasty? J Ophthalmol. (2019) 2019:7813482–7. doi: 10.1155/2019/7813482, PMID: 31482038 PMC6701421

[52] Rocha de Lossada C Airaldi M Semeraro F Romano V. DMEK F-marking complication: case report and literature review. Can J Ophthalmol. (2023) 58:e207–9. doi: 10.1016/j.jcjo.2023.03.011, PMID: 37040868

[53] Veldman PB Dye PK Holiman JD Mayko ZM Sáles CS Straiko MD. The S-stamp in Descemet membrane endothelial Keratoplasty safely eliminates upside-down graft implantation. Ophthalmology. (2016) 123:161–4. doi: 10.1016/j.ophtha.2015.08.044 26439215

[54] Terry MA Straiko MD Veldman PB Talajic JC VanZyl C Sales CS. Standardized DMEK technique: reducing complications using Prestripped tissue, novel glass injector, and sulfur hexafluoride (SF6) gas. Cornea. (2015) 34:845–52. doi: 10.1097/ICO.0000000000000479, PMID: 26075461

